# A Case of COVID-19-Induced Immune Thrombocytopenia (ITP) in an Adult Female: An Under-Recognized Emerging Phenomenon

**DOI:** 10.7759/cureus.38173

**Published:** 2023-04-26

**Authors:** Hayk Ghukasyan, Oksana Petrechko, Horyun Choi

**Affiliations:** 1 Internal Medicine, University of Hawaii, Honolulu, USA; 2 Internal Medicine, University of New Mexico, Albuquerque, USA; 3 Internal Medicine, University of Hawaii Internal Medicine Residency Program, Honolulu, USA

**Keywords:** auto-immune molecular mimicry, 2019 novel coronavirus disease, covid-19, viral itp, itp bleeding, immune thrombocytopenia, covid induced itp, itp in adult, itp managment, covid19 pandemic

## Abstract

Coronavirus disease 2019 (COVID-19) follows a mild course in majority of cases, but some patients may develop non-pulmonary yet life-threatening complications. A Pandora’s box had been opened when multisystem hyper-inflammatory syndromes and autoimmune diseases that had been described previously in children and young adults, that are associated with COVID-19, have now emerged in adults. They need to be recognized as important sequelae of severe COVID-19 disease. Immune thrombocytopenia (ITP) or thrombocytopenic purpura is an autoantibody and T-cell-mediated autoimmune disorder characterized by isolated thrombocytopenia, which can be triggered by different infections. First-line treatment of severe ITP includes platelet transfusions in life-threatening cases, followed by corticosteroids and intravenous immunoglobulins (IVIG). Since the beginning of the pandemic, more and more cases of COVID-19-associated ITP have been reported. We report a case of acquired ITP in a young woman that could only be attributed to her COVID-19 infection and was refractory to platelet transfusion, requiring further treatments. The aim of this report is to review some of the etiologies and purposed molecular mechanisms of the autoimmune nature of the disease and to focus on diagnosis and treatment. We will review the current literature surrounding this non-pulmonary manifestation of COVID-19 and current treatment options for this uncommon presentation of ITP.

## Introduction

Immune thrombocytopenia (ITP) can be a challenging diagnosis and remains a diagnosis of exclusion. It can have life-threatening complications [1]. As with many implicated infectious processes related to ITP, the coronavirus disease 2019 (COVID-19) pandemic has its own hyper-inflammatory profile and autoimmune processes that have come into play and need to be recognized early [2-4]. Syndromes like Kawasaki, toxic shock, and macrophage activation syndrome (MAS) are described in the pediatric and young adult populations and have been associated with COVID-19 [5-8].

As an autoimmune disorder, ITP's widely-accepted pathogenesis is mediated by Fc receptor (FcR) clearance of antibody-opsonized platelets by spleen macrophages [9]. There may also be other unknown mediators of immune dysregulation such as immune complex, cytokine or endothelitis that may be implicated and need to be further elucidated [10,11]. Other potential mechanisms of ITP secondary to COVID-19 include the homology between COVID-19 proteins (33%) and proteins essential to the adaptive immune system, leading to cross-presentation of antigens. Another mechanism includes immune-complex formation on platelet surface via molecular mimicry, as well as the generation of cross-reactive anti-platelet antibodies (anti-GP IIb/IIIa, GP-Ib/IX, or GP-V) that inhibit the development of bone marrow megakaryocytes to promote their apoptosis. Furthermore, direct viral infection plays a role in the expression of cryptic antigens on platelets leading to recognition by the reticuloendothelial system. There may be B and T cell involvement, as well as local cytokine reactions involved in immune dysregulation in a setting of COVID-19 infection [12-14].

The risk of treatment with steroids and intravenous immunoglobulins outweighs the benefits of watchful waiting [3,4]. It has been generally agreed by the scientific community that it is the clinical evidence of bleeding and not the value of the platelet count that drives the rationale for treatment. However, given the life-threatening bleeding risk in a critically low platelet level, the risk-benefit profile favors treatment. Despite the absence of prospective, controlled studies, there is consensus that bleeding risks are significantly greater in patients with platelet counts less than 20-30 x10^9^/L, and therefore treatment is indicated for these patients; for those with platelet counts that are higher, but still, less than 50 x10^9^/L, Treatment is also indicated if there is accompanying substantial mucocutaneous bleeding [15]. The standard initial treatment for ITP is corticosteroids aimed to increase platelet counts. Intravenous immunoglobulin (IVIG) or anti-D immunoglobulin can also increase platelet counts and are particularly useful for stimulating rapid platelet increases before any planned procedures [16]. Ultimately, splenectomy provides a long-lasting response in patients who fail long-term steroid therapy [17]. However, splenectomy is an invasive procedure with some patients relapsing even after several years. Very rare cases of life-threatening or lethal infections may also occur at any time after splenectomy and thus physicians and patients should be vigilant in risk and benefit analysis when choosing to pursue this. The COVID-19 pandemic has unlocked a myriad of rare and unknown clinical presentations of the immune system, and ITP is one such unfortunate manifestation [12].

## Case presentation

We report a case of a 39-year-old woman with a past medical history of well-controlled hypertension and obesity who presented with fever, chills, malaise, cough, and associated nausea and vomiting for five days prior to presentation at the emergency room, where she tested positive for COVID-19. During the hospitalization, she was found to have an isolated progressively declining thrombocytopenia with a nadir of 20,000 cells. On the morning of admission, the patient woke up feeling increasingly short of breath, becoming winded after only walking a few feet. Reportedly, she fell at home and landed on her right hand, but denied loss of consciousness. She endorsed diffuse body aches, malaise, non-bloody, non-bilious vomiting, and decreased oral intake for the past four days. She also endorsed diffuse abdominal pain, decreased appetite, diffuse muscle aches, frontal band-like headache resistant to scheduled acetaminophen, and non-bloody diarrhea for the past few days. She was menstruating with an abnormally large amount of blood soaking pads compared to her usual amount. She denied bleeding in her gums or other bruising anywhere on the body. She had not been vaccinated against COVID-19 due to concern for side effects. Her only home medication per chart review was over-the-counter acetaminophen and ibuprofen as needed for pain, the latter of which she took one month prior (200 mg tablet once twice weekly for occasional headaches). She had not received any heparin-containing products prior to presentation, and her serotonin release assay for heparin-induced thrombocytopenia was negative after heparin prophylaxis in the hospital. She denied any prior history of joint pain, rash, vision changes, hematuria, or known autoimmune diseases in her family. 

Physical examination was remarkable for tachycardia of 101 beats per minute, tachypnea of 22 breaths per minute, elevated systolic and diastolic blood pressures of 160 and 100 mmHg respectively, and normal oxygen saturation on room air. She was a tired-appearing obese woman in moderate respiratory distress. Her lungs were clear to auscultation bilaterally with normal chest rise and without rales or wheezes. Cardiac examination was normal except for tachycardia. Her abdomen was soft yet protuberant, mildly diffusely tender around all four quadrants. She had no hepatomegaly or splenomegaly on examination and endorsed decreased bowel sounds. Her extremities were warm to the touch with only mild pretibial tenderness on the right. Skin examination was normal, with no evidence of purpura but some petechiae noted at ankles. Neurological examination was nonfocal with grossly intact cranial nerves. The patient had a normal and steady gait with clear and fluent speech.

Laboratory values revealed liver enzymes, normal synthetic liver function, and normal basic metabolic profile. The full set of lab workup is summarized in Table 1. Her complete blood count was remarkable for a platelet count of 24,000 cells/mcL that dropped to 20,000 cells/mcL the next day ​(Table 1).

**Table 1:** Complete set of lab values with their corresponding normal levels eGFR CKD-EPI Creatinine 2021, estimated glomerular filtration rate using the Chronic Kidney Disease-Epidemiology Collaboration creatinine equation 2021; HCO3, bicarbonate; SGOT (AST), serum glutamic-oxaloacetic transaminase (aspartate transaminase); SGPT (ALT), serum glutamic-pyruvic transaminase (alanine aminotransferase); INR, international normalized ratio; PT, prothrombin time; PTT, partial thromboplastin time; MCV, mean corpuscular volume; MCH, mean corpuscular hemoglobin; MCHC, mean corpuscular hemoglobin concentration; RDW, red cell distribution width; Abs, absolute; H, high; L, low, ANA, anti-nuclear antibody; HIV, human immunodeficiency virus; EBV, Epstein-Barr Virus; CMV, cytomegalovirus; CRP, C-reactive protein; PCR, polymerase chain reaction; COVID-9, coronavirus disease 2019; hCG: human chorionic gonadotropin

	Patient’s values	Normal reference values
Complete metabolic profile		
Glucose	108 (H)	74 to 110 mg/dL
Blood urea nitrogen	9	8 to 20 mg/dL
Creatinine	0.7	0.4 to 1.5 mg/dL
eGFR CKD-EPI Creatinine 2021	105	≥90 mL/min/1.73 m^2 (60>- )^
Sodium	135	135 to 142 mmol/L
Potassium	3.5	3.5 to 5.2 mmol/L
Chloride	102	100 to 110 mmol/L
HCO_3_	23	20 to 30 mmol/L
Calcium	8.6	8.3 to 10.3 mg/dL
SGOT (AST)	32	<=36 IU/L
SGPT (ALT)	15	
Alkaline phosphatase	51	30 to 120 IU/L
Bilirubin, total	0.9	<=1.0 mg/dL
Total protein	7.2	6.0 to 8.0 gm/dL
Albumin	3.4	3.4 to 5.0 gm/dL
Globulin	3.1	2.1-3.7 g/dL
Albumin/globulin ratio	1.1	1.0-2.4
Complete Blood Count with Differential		
White blood cell count	7.8	3.5 to 10 thousand cells/mL
Red blood cell count	4.44	3.60 to 5.30 million cells/mL
Hemoglobin	14.2	11 to 16 gm/dL
Hematocrit	44.3	33 to 46%
MCV	93.9	82 to 99 fL
MCH	30.6	26 to 34 pg
MCHC	32.6	32 to 36 gm/dL
RDW	13.5	<= 14.5
Platelet count	358	160-420 × 10^3 ^cells/mL
Immature granulocyte	0.3	0.0-1.0%
Neutrophil	45	34.0-72.0%
Lymphocyte	14	12.0-44.0%
Monocyte	9.6	0.0-12.0%
Eosinophil	2.0	0.0-7.0%
Basophil	0.6	0.0-2.0%
Abs immature granulocytes	0.03	0.0-0.10 × 10^3^/mL
Abs neutrophils	4.56	1.56-6.20 × 10^3^/mL
Abs lymphocytes	1.21	1.18-3.74 × 10^3^/mL
Coagulation Studies		
PT	15.0 (H)	11.4-15.2 sec
INR	0.9	<= 3.0
PTT	37.8	26 to 38
Special Tests		
COVID-19 PCR	Detected	Undetectable
Influenza A/B PCR	Not detected	Not detected
CRP	<3	0.0 to 10.0 mg/L
B-hCG Qualitative	Negative	Negative
Folate level	76 H	>3.1 ng/mL
Vitamin B12 level	495	232-1245 pg/mL
ANA	<40	<=40
C3	121	90-280 mg/dL
C4	62 H	10-40 mg/dL
Anti-Cardiolipin Ab IgG	7.5	<20.0 GPL-U/mL
Anti-Cardiolipin Ab IgM	0.4	<20.0 MPL-U/mL
Anti-B1-Glycoprotein 1 IgG Ab	<1.4	<20.0 Units/mL
Anti-B1-Glycoprotein 1 IgM Ab	0.5	<20.0 Units/mL
Hepatitis B Virus	Negative	Negative
Hepatitis C Virus	Negative	Negative
Anti-Platelet Circulating Antibodies IgG	Negative	Negative
Anti-Platelet Circulating Antibodies IgM	Positive	Negative
HIV	Negative	Negative
EBV	negative	Negative
CMV	Negative	Negative
Hemoglobin A1c	5.9%	<5.7 %

**Figure 1:**
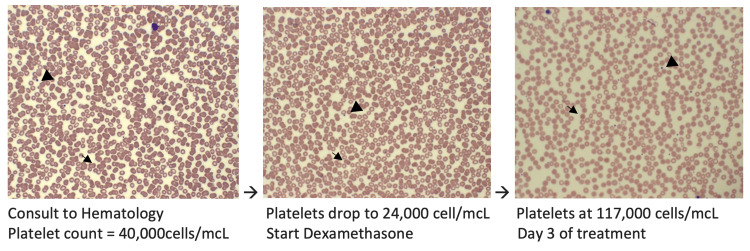
Serial peripheral smears revealing initial smear with decreased platelets without clumping, following treatment with improved platelet counts (arrowhead), in a background of red blood cells (arrow).

Reverse transcriptase PCR assay detected the presence of severe acute respiratory syndrome coronavirus 2 (SARS-CoV-2) RNA in the nasopharyngeal swab. The patient had negative troponins and normal basic metabolic profile. She had normal vitamin B12 and folate levels, white blood cells and hemoglobin were within normal limits. Anti-Platelet circulating Ab IgM was positive, IgG was negative. Beta-2 Glycoprotein 1 IgG and IgM were within normal limits. and Cardiolipin antibodies were within normal limits. Her coagulation studies were normal. Her Influenza A and B, HIV, Epstein-Barr virus (EBV), cytomegalovirus (CMV), and hepatitis C virus (HCV) statuses were negative, and she was immune to hepatitis B virus. Given her ongoing headache, CT scan of the head was done which ruled out an intracranial bleed. Her peripheral blood smear revealed true thrombocytopenia without clumping and without evidence of schistocytes to suggest thrombotic microangiopathy (Figure 1).

**Figure 2:**
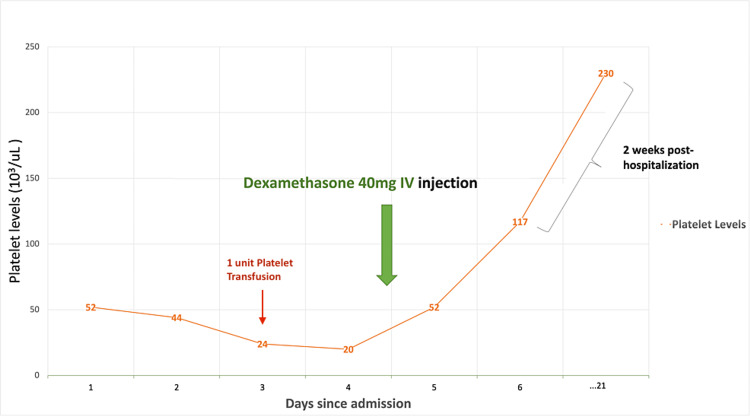
The time course of platelet counts to nadir and improvement of our patient, who underwent platelet transfusion and dexamethasone treatment. Red arrow indicates the day of one unit of platelet transfusion. Green arrow indicates the commencement of dexamethasone treatment intravenously, given 40 mg/day for four days.

Hematology service was consulted, and a decision was made to treat the patient with intravenous pulse dose steroids given her poor oral intake and ongoing nausea and vomiting. The aim was to limit prolonged steroid exposure. If thrombocytopenia and bleeding persisted, the plan was to start IVIG treatment. The patient received dexamethasone 40mg IV daily for four days, and platelet counts started to improve, as well as her headache, nausea, and menstrual bleeding. There was no need for IVIG treatment. After only two days of pulse dose steroids, platelet counts improved to 117,000 cells and eventually were normalized after the complete course (Figure 2).

## Discussion

ITP is an acquired disorder characterized by peripheral destruction of platelets as a result of loss of primarily or secondarily induced immune tolerance [18]. Platelets coated with IgG autoantibodies undergo accelerated clearance through Fcy receptors that are expressed by tissue macrophages, predominantly in the spleen and liver. COVID-19-mediated ITP can be attributed to the underlying immune dysregulation, susceptibility mutations, and other mechanisms including molecular mimicry, cryptic antigen expression, and epitope spreading, possibly driven by the generation of cross-reactive antibodies to glycoproteins on platelet surfaces [19].

As in many other autoimmune diseases, some viruses have been identified as a trigger for the immunological activation of ITP. For instance, immune system stimulation through antigens via “molecular mimicry” has been well described with varicella-zoster viruses, HIV, HCV, and *Helicobacter pylori* bacterium [20]. A similar mechanism can be an underlying mechanism in COVID-19 since platelets are increasingly recognized as active players in the (antiviral) immune responses and have been shown to interact with cells of the innate and adaptive immune system as well as directly with viruses [20].

A recent systematic review by Bhattacharjee et al. looked at the clinical profile and outcomes in more than 40 cases of new-onset ITP in COVID-19 patients, necessitating a comprehensive approach is essential for diagnosing COVID-19-associated ITP and prompt treatment [12]. To this end, it is important to exclude other concomitant factors that can cause thrombocytopenia in COVID-19 infection. For instance, there have been studies looking at drug-dependent antibodies to acetaminophen and ibuprofen [21].

For acetaminophen, drug-dependent antibodies have been identified only for metabolites and do not react with the unmodified drug. For ibuprofen, drug-dependent antibodies that reacted with the unmodified drug have been identified in some patients; in other patients, drug-dependent antibodies only reacted with metabolites of ibuprofen. Given that our patient took a low dose of ibuprofen and acetaminophen more than a month ago and only twice weekly for the occasional headache, the hematology team thought that this was very unlikely to be the cause of the thrombocytopenia and no further studies of drug-dependent antibodies were pursued given low diagnostic yield and increased cost with no changes in management [22]. During the hospitalization, the patient required only a one-time dose of 500 mg oral dose of acetaminophen for headache on the day of discharge. Nonetheless, drug-induced ITP (DITP) is often suspected in patients with acute thrombocytopenia unexplained by other causes and remains part of the differential diagnosis. 

It is known that COVID-19 can affect the hematopoietic system. We know that in patients with chronic ITP, thrombocytosis can be observed after COVID-19 infection, frequently needing treatment adjustment or even discontinuation of therapy [23]. However, a decrease in platelets in a setting of COVID-19 infection is a paradoxical phenomenon in this respect. Moreover, treatment of severe COVID-19-associated ITP in critically ill patients may be particularly challenging because of side effects, detrimental effects of steroids on immune function, and concerns for poor viral clearance from corticosteroids. Overall outcomes of treatment for COVID-19-associated ITP with IVIG and steroids have yet to be elucidated; thus all cases must continue to be reported to add to our current understanding of clinical manifestations and evaluation of treatment strategies [24].

## Conclusions

Thrombocytopenia is a risk factor for increased morbidity and mortality in patients infected with COVID‐19. The criteria and treatment options for ITP and the benefits-risks balance of such therapies were discussed in this report. Ongoing and future studies will help define the best strategies for increasing platelet counts and reducing the risk of bleeding in ITP patients. In addition to cross-linking and molecular mimicry, other possible contributing mechanisms of COVID-19-mediated thrombocytopenia have already been described. These mechanisms can involve inhibition of platelet synthesis via direct infection of the bone marrow cells or platelets by the virus and dysfunctional marrow microenvironment; virus-mediated liver damage leading to decreased thrombopoietin (TPO) production, pulmonary endothelial damage followed by platelet aggregation in the lungs, subsequent formation of microthrombi, and platelet consumption. In addition, the destruction of platelets by the immune system manifested as a dramatic fall from baseline. 

It is important to be aware of this severe complication of a COVID‐19 infection and should be diagnosed and treated immediately. Timely recognition is critical as otherwise it may ultimately lead to fatal complications such as intracranial hemorrhage. The choice of treatment for ITP should be based on balancing the risk of bleeding due to ITP versus the potential deterioration of COVID‐19 infection due to immunosuppression from steroid therapy.
